# Anomaly detection in radiotherapy plans using deep autoencoder networks

**DOI:** 10.3389/fonc.2023.1142947

**Published:** 2023-03-14

**Authors:** Peng Huang, Jiawen Shang, Yingjie Xu, Zhihui Hu, Ke Zhang, Jianrong Dai, Hui Yan

**Affiliations:** Department of Radiation Oncology, National Cancer Center/National Clinical Research Center for Cancer/Cancer Hospital, Chinese Academy of Medical Sciences and Peking Union Medical College, Beijing, China

**Keywords:** treatment plan, unsupervised learning, autoencoder, detection, radiotherapy

## Abstract

**Purpose:**

Treatment plans are used for patients under radiotherapy in clinics. Before execution, these plans are checked for safety and quality by human experts. A few of them were identified with flaws and needed further improvement. To automate this checking process, an unsupervised learning method based on an autoencoder was proposed.

**Methods:**

First, features were extracted from the treatment plan by human experts. Then, these features were assembled and used for model learning. After network optimization, a reconstruction error between the predicted and target signals was obtained. Finally, the questionable plans were identified based on the value of the reconstruction error. A large value of the reconstruction error indicates a longer distance from the standard distribution of normal plans. A total of 576 treatment plans for breast cancer patients were used for the test. Among them, 19 were questionable plans identified by human experts. To evaluate the performance of the autoencoder, it was compared with four baseline detection algorithms, namely, local outlier factor (LOF), hierarchical density-based spatial clustering of applications with noise (HDBSCAN), one-class support vector machine (OC-SVM), and principal component analysis (PCA).

**Results:**

The results showed that the autoencoder achieved the best performance than the other four baseline algorithms. The AUC value of the autoencoder was 0.9985, while the second one was 0.9535 (LOF). While maintaining 100% recall, the average accuracy and precision of the results by the autoencoder were 0.9658 and 0.5143, respectively. While maintaining 100% recall, the average accuracy and precision of the results by LOF were 0.8090 and 0.1472, respectively.

**Conclusion:**

The autoencoder can effectively identify questionable plans from a large group of normal plans. There is no need to label the data and prepare the training data for model learning. The autoencoder provides an effective way to carry out an automatic plan checking in radiotherapy.

## 1 Introduction

Cancer treatment has significantly progressed with the development of technology in recent years. Because radiotherapy plays an important role in cancer treatment, it has been increasingly used. At present, radiotherapy (RT) is an indispensable treatment for nearly all cancer types. Approximately 60% of cancer patients receive RT for neoadjuvant, definitive, adjuvant, or palliative purposes. Furthermore, among all patients who survive malignant tumors, up to 40% have been cured by RT alone or in combination with other modalities (1). As a treatment method using radiation, radiotherapy requires a higher accuracy to ensure the safety of treatment. It delivers a higher radiation dose to the target area of a patient and deposits a dose as low as possible to the surrounding healthy tissues. This will significantly damage the tumor tissue in the target area while protecting the normal tissue from unnecessary irradiation. It also means that any small mistakes caused by the clinician, treatment planner, and machine operator will pose a large risk to patients.

Given the complexity of modern radiotherapy, stricter policies are needed to ensure consistency between the delivered and calculated plans for the increasing quality and safety requirements (2). Therefore, extreme caution and enhanced quality control are necessary to ensure the safety of radiotherapy. Physicians and radiation physicists conduct independent reviews of plans before and after treatment to ensure that the plans meet hospital standards (3). The physics plan and the chart check are the most effective quality control methods for reducing human error in radiotherapy (4). A full treatment plan review involves diagnosis, prescription, planning, and approximately 20 field-specific parameters, and newer radiation techniques often bring in even more parameters (5). Each of these parameters can affect treatment efficacy and patient safety. A full human review would make the plan review entirely dependent on the reviewer’s experience, and even an experienced human reviewer could accidentally miss the errors (6).

Compared with manual plan checking, automatic plan checking can save manpower and also alleviate the miscalculation caused by fatigue. Therefore, it is widely adopted in clinical practice to partially or completely replace the manual reviewing process. A semi-automatic system called AutoLock was proposed for radiotherapy plan quality control (QC) (7). It aims to enhance the quality control of the treatment plan through automatic inspection. The Plan-Checker Tool (PCT) was proposed, which uses an application programming interface to check and compare radiotherapy plan data (8). The semi-automatic method increases the visibility of errors during physical examination, thus reducing patient delay. Furhang et al. developed a software to automatically carry out planned and interplanned reviews (9). Yang et al. conducted a study to automatically verify the integrity of the treatment plan using dynamic scripts (10).

As a popular structure of the deep neural network, the autoencoder has been widely used in different fields, including shape representation (11) and image segmentation (12). The autoencoder was also introduced in anomalous data analysis, and its capability has also been demonstrated (13–16). Schreyer et al. used an autoencoder to detect anomalous accounts in bank accounting data (17). Meidan et al. proposed an algorithm called N-BaIoT to detect attacks launched from IoT devices and to distinguish between IoT-based attacks lasting for hours and milliseconds (18). It was also used for unsupervised sound anomaly detection to detect unknown abnormal sounds without abnormal sound training data (19).

Recently, the autoencoder has been increasingly investigated in radiotherapy. Mezheritsky et al. developed a population-based 3D respiratory motion modeling from convolutional autoencoders for 2D ultrasound-guided radiotherapy (20). The model was trained on a variety of deformations and anatomies which enable it to generate the 3D motion experienced by the liver of a previously unseen subject. Dou et al. proposed a predictive maintenance framework based on a long short-term memory-based autoencoder to detect rare anomalous machine events for a proton delivery system (21). These included QA beam pauses, clinical operational issues, and treatment interruptions. Wang et al. developed a novel multitask model called autoencoder-based classification-regression for volumetric-modulated arc therapy (VMAT) of patient-specific QA (22). The model was later commissioned and implemented in multi-institution scenarios (23).

For plan checking, there are still no such applications due to the limited training data and the complexity of the plan parameters. In this study, the autoencoder was introduced to identify the questionable plan from the regular plans based on the magnitude of the reconstruction error. The larger reconstruction error indicated the outlier from the central distribution of standard plans. The rest of this manuscript is organized as follows. In Section 2, the patient data and algorithm were explained. In Section 3, the performance of the autoencoder was analyzed and compared with other traditional detection algorithms. In Section 4, the advantages and disadvantages of the proposed method were discussed.

## 2 Materials and methods

### 2.1 Data

Five hundred seventy-six radiotherapy plans for breast cancer patients were collected in our institute. Among these, 557 were “normal” plans and 19 were “abnormal” plans identified by human experts. All these plans were clinically approved for treatment. The normal plans are those plans which completely meet our treatment goals and have no flaws. The abnormal plans are those plans which also completely meet our treatment goal but have certain flaws in plan settings and parameters. The abnormal plans are not error plans but are hardly detected by a rule-based plan-checking procedure. For example, the institutional protocol may require that five to seven equally spaced beams should be included in a plan. If a plan contains beams less than 5 or the beam spacing is unequal, it would be regarded as an abnormal plan and requested for further improvement by a plan checker. Potentially, these abnormal plans have higher risks to cause errors during treatment.

The radiotherapy plan consists of two conventional tangent fields and two intensity-modulated radiation therapy (IMRT) fields. To characterize these plans, 30 features were extracted and shown in **Table 1**. The number of segments of the IMRT field was the number of apertures through which the radiation dose is delivered. The radiation dose was measured by the monitor unit (MU). The higher the MU value, the higher the dose. The dose was 290 MU for the IMRT field and 200 MU for the tangent field. The other features, such as collimator position, collimator angle, gantry angle, and MU per field, are mechanical parameters related to the radiation devices. For each field, these parameters were varied.

**Table 1:** Description of the features extracted from the treatment plans.

Features	Description	Fields	Number of features	Type	Unit
Segment	The number of segments of the field	IMRT	2	Integer	Number
SSD	Source to skin distance	IMRT/tangent	4	Float	cm
Collx1	Collimators’ position in the x1 direction	IMRT/tangent	4	Float	cm
Collx2	Collimators’ position in the x2 direction	IMRT/tangent	4	Float	cm
Colly1	Collimators’ position in the y1 direction	IMRT/tangent	4	Float	cm
Colly2	Collimators’ position in the y2 direction	IMRT/tangent	4	Float	cm
*G_θ_*	The angle of the gantry	IMRT/tangent	4	Integer	Degree
Meterset	The MU per field	IMRT/tangent	4	Float	MU

According to the types of these features, proper preprocessing was performed to prepare the data before they can be fitted to a learning model. The features in continuous variables, such as the radiation dose, were kept as originally obtained. The features in categorical variables, such as the type of field, were mapped to integers by the one-hot encoding technique. One-hot encoding is a method that converts categorical data into integers. With one-hot encoding, each categorical value is converted into a new categorical column, and a binary value of 1 or 0 is assigned to those columns. Each integer value is represented as a binary vector. All the values are zero, and the index is marked with 1. After one-hot encoding, the original feature dimensions were extended from 30 to 58.

### 2.1 Autoencoder

**Figure 1:**
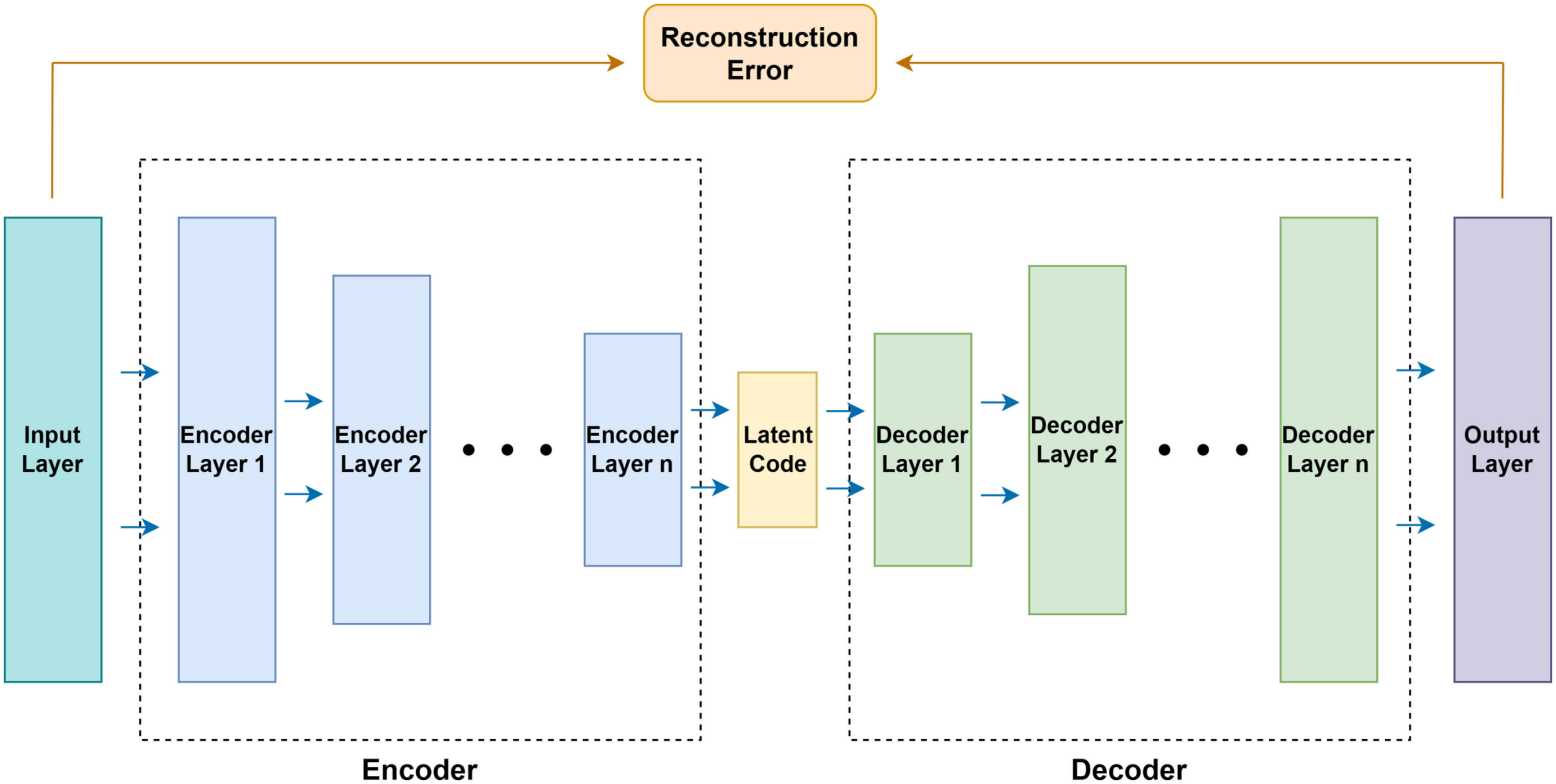
The structure of the autoencoder.

The autoencoder defines a feedforward multilayer neural network with bottlenecks of conformational symmetry as shown in **Figure 1**. First, the data flows go through multiple successive compression layers and then go through multiple successive expansion layers. The loss of the network is the error between the input and the output layers, called the reconstruction error. The goal of learning is to train the network to reconstruct the input of the network. The autoencoder captures the salient features and removes the correlated features by mapping the input data to a high-dimensional space through the bottleneck structure.

The autoencoder consists of an encoder and a decoder. Both the encoder and the decoder contain multiple consecutive basic blocks. Each basic block contains a pair of fully connected layer and activation function, which can be expressed as *f*(*x*|*θ*)=*σ*(*W*·*x*+*B*). Here, *θ* is the parameter of the layer, *W*∈*R* ^*d*_*o*_^×*R* ^*d*_*i*_^ is the weight of the fully connected layer, *B*∈*R* ^*d*_*o*_^ is the bias of the fully connected layer, and *σ* is the non-linear leaky ReLU activation. Also, the dropout layer after each activation function except the output layer was introduced to prevent overfitting of the network.

The fully connected layer contained in the encoder gradually decreases in dimensionality and finally generates the layer with the lowest dimensionality, called the latent code. On the other hand, the fully connected layer contained in the decoder gradually increases in dimensionality and recovers the latent code generated by the encoder to the same dimensionality as the network input layer. Thus, the autoencoder is denoted as $\hat{X}=D\left(E\left(X,\;\theta_{E}\right),\;\theta_{D}\right)$ , where *E*(·,*θ*_*E*_) and *D*(·,*θ*_*D*_) are the encoder and the decoder, and *θ_E_* and *θ_D_* are the parameters of the encoder and decoder. In order to achieve $\hat{X}\approx X$ , the autoencoder needs to be trained to reduce the difference between the treatment plan *X* and the network reconstruction $\hat{X}=D\left(E\left(X,\;\theta_{E}\right),\;\theta_{D}\right)$ . Therefore, the training objective of the network is to find the appropriate parameter *θ* to make the reconstruction error *L_recon_* of the network as small as possible, so the optimization goal of the network can be expressed as:


$$\arg\underset{{\text{θ}}_{\text{E}},\theta_{D}}{\min}L_{recon}\left(X,\;D\left(E\left(X,\;\theta_{E}\right),\;\theta_{D}\right)\right)$$

To enable the autoencoder to capture changes in enumerated and discrete variables after one-hot encoding, a binary cross-entropy function is used to penalize the reconstruction errors.

Also, in order for the autoencoder to capture changes in continuous variables, the mean square loss function is used to penalize the reconstruction error.

They can then be weighted by parameter *λ* and form the overall loss function of the autoencoder:


$$L_{recon}=L_{BCE}+\lambda L_{MSE}$$

The depth of the hidden layers was set to 3 and *λ* was 0.99 based on our experiments. The Adam optimizer is adopted, with a learning rate of 1e−3.

### 2.2 Anomaly detection

In this study, the reconstruction loss distribution of all data is used to select a predefined threshold. Samples are judged as normal or abnormal plans based on their distance from the reconstruction loss threshold. If this distance is greater than the preset threshold, this plan is considered abnormal; otherwise, it is normal. The optimal threshold is chosen to ensure a low false-positive rate (FPR, i.e., judging a point as abnormal when it is actually normal) and a high true-positive rate (TPR, i.e., judging a point as abnormal when it is actually abnormal). Because radiation damage is irreversible in patients, the goal of plan checking is to ensure that all abnormal plans are detected (TPR==1) and the percentage of wrongly detected abnormal plans (FPR) is as low as possible. Therefore, in this study, TPR==1 was required to make sure all abnormal plans were detected. The other performance metrics were evaluated while this condition was satisfied.

### 2.3 Evaluation

The performance of the anomaly detection algorithm was evaluated based on the receiver operating characteristic curve (ROC) and the area under the receiver operating characteristic curve (AUC). The ROC evaluates the ability of the model to distinguish between abnormal and normal plans when a preset threshold is changed. The AUC value is the area under the ROC curve. The larger the AUC value, the better the algorithm itself can distinguish abnormal plans. In addition, considering the highly unbalanced distribution of abnormal and normal classes in the dataset, the accuracy [(true positive + true negative)/(true positive + false positive + true negative + false negative)], precision (true positive/(true positive + false positive)), and F1 score (2 * precision * recall/(precision + recall), where recall = true positive/(true positive + false negative)) of the model were calculated to comprehensively evaluate the performance. A higher value means that the algorithm has a higher ability to make anomalous judgments. Also, anomalies report the number of data judged as abnormal by the algorithm.

The proposed anomaly detection algorithm was evaluated in two aspects. 1) Whether the autoencoder architectures with different layer depths are able to learn the distribution of normal plans: to evaluate the influence of network architectures, autoencoders with six different layer depths from 1 to 6 were trained, and the experiments were repeated 10 times. 2) Whether the autoencoder performance is better compared with the other classical anomaly detection algorithms: for this purpose, four popular detection algorithms were tested, namely, local outlier factor (LOF) (24), hierarchical density-based spatial clustering of applications with noise (HDBSCAN) (25), one-class support vector machine (OC-SVM) (26), and principal component analysis (PCA) (27). LOF is a density-based detection algorithm and HDBSCAN is a clustering-based algorithm. OC-SVM uses a single classification algorithm based on the optimization algorithm. PCA calculates the reconstruction loss of linear mapping.

## 3 Results

### 3.1 The number of layers

**Figure 2:**
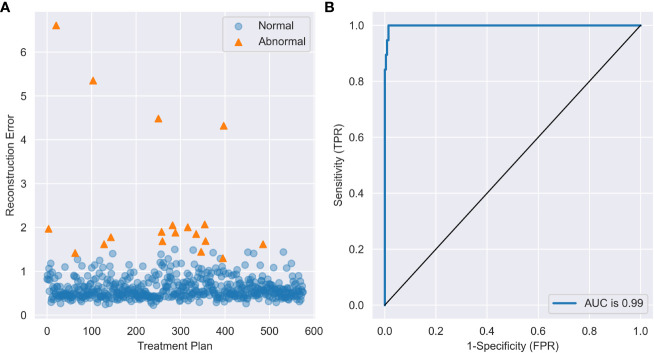
**(A)** The reconstruction error distribution of all data and **(B)** the ROC plot with AE6.

As shown in **Table 2**, autoencoder (AE) 6 achieved an average AUC value of 0.9947 with a maximum of 0.9986. While maintaining 100% recall, the average values of accuracy and precision reached 0.9658 and 0.5143, respectively, and the maximum values reached 0.9844 and 0.6923. This result showed that the depth of the autoencoder has certain effects on its ability to model the inherent manifold structure. The reconstruction error distribution and its corresponding ROC plot using AE6 are shown in **Figure 2**.

**Table 2:** Comparison of the autoencoder with different encoder layer depths.

	AUC (max)	Accuracy (max)	Precision (max)	FPR (min)	F1 score (max)	Anomalies (min)
AE9 (depth = 9)	0.93 (0.99)	0.79 (0.97)	0.21 (0.60)	0.21 (0.02)	0.33 (0.73)	139.70 (30)
AE8 (depth = 8)	0.98 (0.99)	0.90 (0.98)	0.35 (0.66)	0.10 (0.01)	0.49 (0.78)	75.50 (27)
AE7 (depth = 7)	0.98 (0.99)	0.93 (0.98)	0.41 (0.69)	0.07 (0.01)	0.56 (0.79)	58.70 (26)
AE6 (depth = 6)	0.99 (0.99)	0.96 (0.98)	0.51 (0.69)	0.03 (0.01)	0.67 (0.80)	38.50 (26)
AE5 (depth = 5)	0.99 (0.99)	0.95 (0.98)	0.44 (0.63)	0.04 (0.01)	0.60 (0.77)	45.80 (30)
AE4 (depth = 4)	0.98 (0.99)	0.94 (0.97)	0.39 (0.61)	0.05 (0.02)	0.55 (0.76)	52.20 (31)
AE3 (depth = 3)	0.97 (0.99)	0.90 (0.97)	0.90 (0.97)	0.10 (0.02)	0.47 (0.71)	74.80 (34)
AE2 (depth = 2)	0.95 (0.99)	0.88 (0.96)	0.26 (0.46)	0.11 (0.03)	0.40 (0.63)	84.80 (41)
AE1 (depth = 1)	0.94 (0.99)	0.84 (0.94)	0.20 (0.39)	0.16 (0.05)	0.33 (0.56)	109.10 (48)

The max and min indicated the maximal and minimal values of statistics in 10 tests.

**Figure 3:**
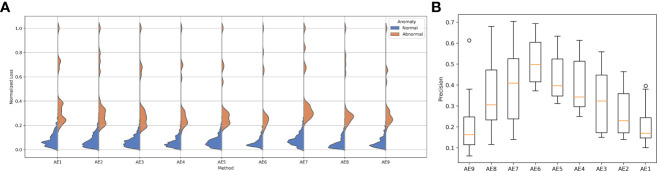
**(A)** Reconstruction error distribution for the autoencoder with different layers and **(B)** the precision distribution of 10 repeated experiments for the autoencoder with different layers.

The reconstruction error distribution using six different autoencoders with layer depths from 1 to 6 is illustrated in **Figure 3A**. AE6 showed the lowest overlap between the reconstruction error distribution of abnormal and normal data. **Figure 3B** illustrates the precision distribution achieved by the autoencoders with different layer depths after repeating the experiment 10 times. AE6 achieved the highest precision overall.

**Figure 4:**
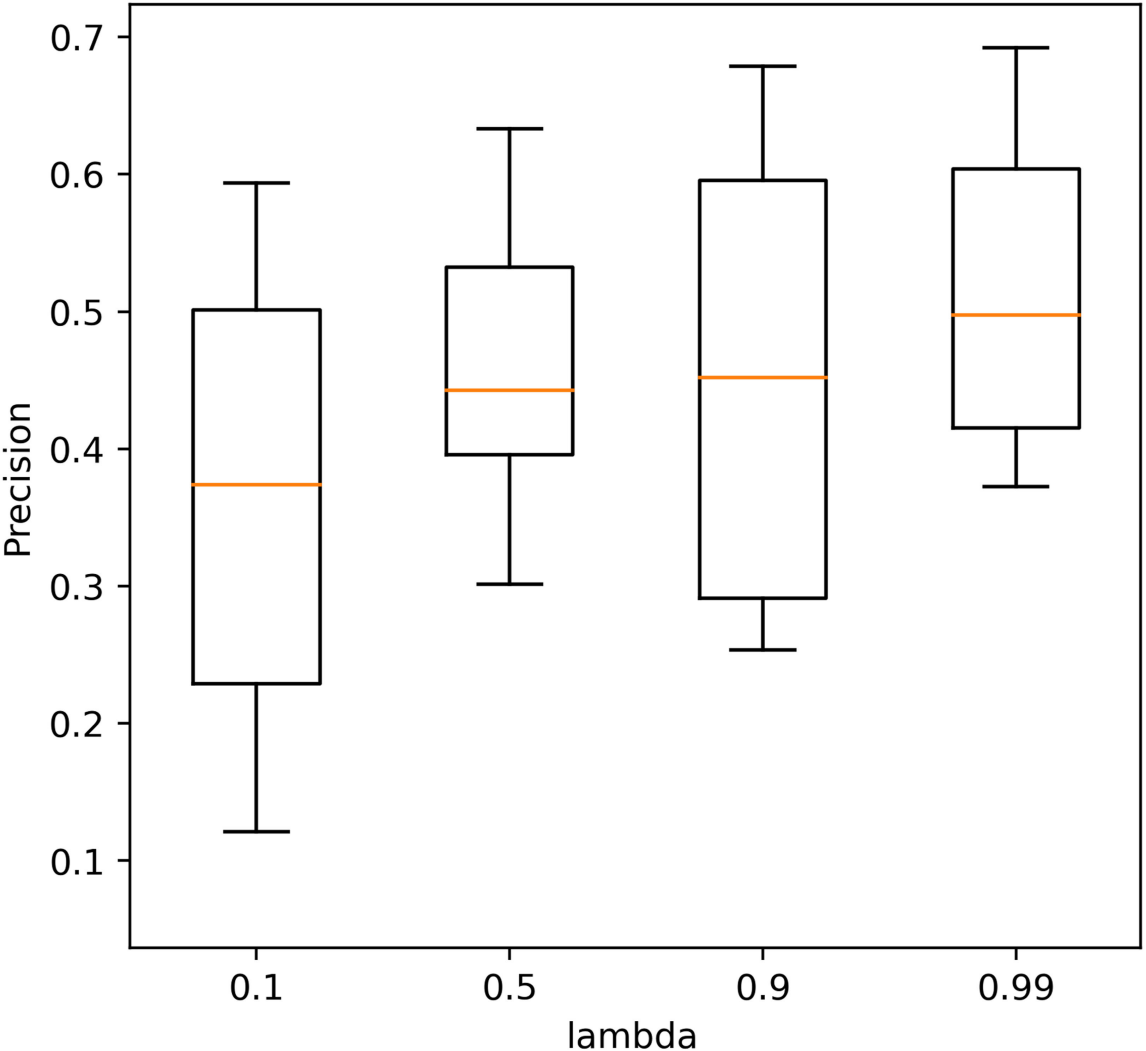
The precision of the autoencoder with different *λ* values in 10 repeated tests.

Four different values (0.1, 0.5, 0.9, 0.99) of *λ* were tested to determine its optimum value. The precision of the AE with different *λ* values in 10 repeated tests is shown in **Figure 4**. The performance with a *λ* value of 0.99 shows the highest precision, and the performance with a *λ* value of 0.9 is closer but with a larger standard deviation. The average precision caused by different *λ* values is within [0.38, 0.48] which also shows less effect of *λ* on the performance of the AE.

### 3.2 Baseline model evaluation

To compare each algorithm as fairly as possible, we used a grid-search algorithm to search for the parameters in each algorithm that would achieve the best detection results. **Table 3** shows the anomaly detection performance of the autoencoder and the traditional detection algorithms, and the corresponding best parameters are also listed in the table. The autoencoder algorithm achieved the best anomaly detection performance than the other benchmark techniques. Compared with the LOF algorithm, which has the best results among the traditional algorithms, the autoencoder algorithm achieved a substantial improvement in all metrics, and the accuracy and precision have been improved up to 0.1753 and 0.5451.

**Table 3:** Comparison of the four baseline detection algorithms.

	AUC	Accuracy	Precision	FPR	F1 score	Anomalies
AE (depth = 6)	0.99	0.98	0.69	0.01	0.79	26
LocalOutlierFactor (n_neighbor = 10)	0.95	0.80	0.14	0.19	0.25	129
HDBSCAN (min_cluster_size = 200)	0.91	0.76	0.12	0.63·	0.21	155
OneClassSVM (nu = 0.001, gamma = 0.1)	0.70	0.19	0.03	0.83	0.07	483
PCA (*n* = 36)	0.83	0.47	0.05	0.54	0.11	321

**Figure 5:**
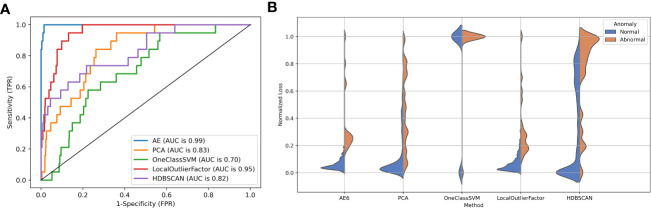
**(A)** The ROC curve and **(B)** reconstruction error distribution using the autoencoder and traditional methods.

**Figure 5A** shows the ROC performance of the different detection algorithms. The autoencoder outperformed the traditional algorithm because it is closer to the upper left corner of the figure. **Figure 5B** shows the distribution of reconstruction errors using different detection algorithms. The autoencoder had the lowest overlap between the reconstruction error distributions for abnormal and normal data.

## 4 Discussion

This study evaluates the performance of autoencoders in determining abnormal data and comparatively investigates the performance differences between autoencoder networks and a variety of commonly used traditional anomaly detection algorithms. The results show that the autoencoder is superior to traditional anomaly detection algorithms in several aspects. First, the AE method employs a non-linear activation function in the encoder/decoder, allowing the neural network to arbitrarily approximate any non-linear function. This allows the network to learn more complex mapping relationships between high-dimensional space and low-dimensional space, to better fit the distribution of normal data, and thus, to find abnormal data with a very small percentage through the network. Second, the autoencoder-based algorithm can effectively separate normal and abnormal data in the reconstructed error distribution map in anomaly detection, showing higher accuracy and precision. The ROC and AUC scores also show that the AE-based model outperforms the traditional anomaly detection algorithm. Third, traditional anomaly detection algorithms are less flexible and only have a few parameters for model tuning. The network structure of the autoencoder can be adjusted easily according to specific tasks. It can be trained according to different tasks and data, which has a higher task specificity.

The experimental results showed that the autoencoder with six layers of encoders and decoders had the best performance. With layers of more than six, the excessive number of neurons increases the training cost and causes overfitting of the model. This was demonstrated in AE9, whose accuracy and precision decreased significantly compared with those of AE6. On the contrary, if fewer layers are used, the model does not have enough space to learn the data and the performance of the model is also compromised. This was demonstrated in AE1, whose accuracy and precision decreased considerably compared with those of AE6. Therefore, the proper selection of layers is important for model performance. It would be possible to search for these hyperparameters using grid-search techniques.

Common to most clinical scenarios, the rate of an abnormal plan is very low, and an error plan (e.g., wrong beam energy or beam angles) is even rare. A challenge to this study is the detection rate of anomalous events in a highly imbalanced dataset. Hence, model performance was evaluated primarily using the precision–recall metrics. Numerous studies have shown that AUC may misrepresent model performance, especially in imbalanced datasets (28, 29). Since precision–recall metrics focus on the model performance of the positive or minority class, they are particularly suitable for our problem. In this study, TPR (recall) was set to 1 to make sure all abnormal plans were detected. Then, the other performance metrics were evaluated under this initial constraint. In addition to AUC and precision, the accuracy and F1 score were provided to present a comprehensive evaluation of these anomaly detection models.

The application of the AE-based approach in the detection of abnormalities in radiotherapy treatment protocols is promising. However, it can be improved in several aspects. First, the raw features extracted from the plans are mainly based on the expert’s experience rather than quantified. Due to the inconsistency of plans, some features that may be sensitive to abnormal plans are not included in this study. Second, as a special configuration of deep neural networks, autoencoders are also similarly black box in nature, making further analysis and interpretation of their results difficult for the time being. This may be improved in the future by introducing some interpretable network structures. Third, the AE model used in this study is a simple model, and most of the model parameters are not optimized for specific tasks. With a better model and optimized parameters, the performance of the AE could be further improved. It could be learned by certain optimization techniques such as neuroevolution (30–32), which is scalable and efficient.

For valid inputs, the AE is able to compress them to fewer bits, essentially getting rid of the redundancy (encoder), but due to the non-regularized latent space in the AE, the decoder cannot be used to generate valid input data from latent vectors sampled from the latent space. The variational autoencoder (VAE) addresses the issue of non-regularized latent space in the autoencoder and provides the generative capability to the entire space. The encoder in the AE outputs latent vectors. Instead of outputting the vectors in the latent space, the encoder of the VAE outputs parameters of a predefined distribution in the latent space for every input. The VAE then imposes a constraint on this latent distribution forcing it to be a normal distribution. This constraint ensures that the latent space is regularized and precisely controlled. This makes the VAE more practical and feasible for large-scale datasets. Therefore, it would be our next work to implement VAE for anomaly detection in plan checking of radiotherapy.

## 5 Conclusion

The autoencoder provides an anomaly detection algorithm for radiation treatment planning. It can detect a very small percentage of abnormal plans in a large number of radiotherapy plans with high accuracy and precision. Our evaluation using a real radiotherapy treatment plan dataset shows that the autoencoder-based detection algorithm can achieve an AUC value of 0.9985, which is a maximum improvement of 41.77%, compared with the traditional anomaly detection algorithm. Given the large number of radiotherapy plans generated by the department on a daily basis, this algorithm will save significant time for reviewers and reduce the risk of low-quality plans.

## Data availability statement

The datasets presented in this article are not readily available because include participant identifiable data. Requests to access the datasets should be directed to cukyhuang@163.com.

## Author contributions

Conception and design: PH and JS. Administrative support: JD and HY. Provision of study materials or patients: YX. Collection and assembly of data: ZH and KZ. Data analysis and interpretation: PH and JS. All authors contributed to the article and approved the submitted version.
